# Metabolism and immune memory in invertebrates: are they dissociated?

**DOI:** 10.3389/fimmu.2024.1379471

**Published:** 2024-07-11

**Authors:** Texca T. Méndez-López, Julio César Carrero, Humberto Lanz-Mendoza, Alejandra Ochoa-Zarzosa, Krishnendu Mukherjee, Jorge Contreras-Garduño

**Affiliations:** ^1^ Posgrado en Ciencias Biológicas, Escuela Nacional de Estudios Superiores, Unidad Morelia, Universidad Nacional Autónoma de México, Morelia, Mexico; ^2^ Departmento de Immunología, Instituto de Investigaciones Biomédicas, Universidad Nacional Autónoma de México, Ciudad de México, Mexico; ^3^ Instituto Nacional de Salud Pública, Departamento de Enfermedades Infecciosas, Cuernavaca, Mexico; ^4^ Centro Multidisciplinario de Estudios en Biotecnología, Universidad Michoacana de San Nicolás de Hidalgo, Morelia, Mexico; ^5^ Institute of Hygiene, University Hospital Müenster, University of Münster, Münster, Germany; ^6^ Institute for Evolution and Biodiversity, University of Münster, Münster, Germany; ^7^ Escuela Nacional de Estudios Superiores, unidad Morelia, Universidad Nacional Autónoma de México, Morelia, Mexico

**Keywords:** immunometabolism, specific memory, ecoimmunology, host-parasite relationship, innate immune response, immune priming, trade-offs

## Abstract

Since the discovery of specific immune memory in invertebrates, researchers have investigated its immune response to diverse microbial and environmental stimuli. Nevertheless, the extent of the immune system’s interaction with metabolism, remains relatively enigmatic. In this mini review, we propose a comprehensive investigation into the intricate interplay between metabolism and specific immune memory. Our hypothesis is that cellular endocycles and epigenetic modifications play pivotal roles in shaping this relationship. Furthermore, we underscore the importance of the crosstalk between metabolism and specific immune memory for understanding the evolutionary costs. By evaluating these costs, we can gain deeper insights into the adaptive strategies employed by invertebrates in response to pathogenic challenges. Lastly, we outline future research directions aimed at unraveling the crosstalk between metabolism and specific immune memory. These avenues of inquiry promise to illuminate fundamental principles governing host-pathogen interactions and evolutionary trade-offs, thus advancing our understanding of invertebrate immunology.

## Graphical Abstract

Immune and metabolic parameters reported in studies of immune memory in invertebrates. After **(A)** the priming challenge is observed, metabolic activation, epigenetic markers, endoreplication, and differential immune effector mechanisms (e.g. antimicrobial peptides (AMPs) or phagocytosis) are observed. After **(B)** the second challenge, metabolic, epigenetic, endocycling, and immune effector mechanisms are also activated. Whether similar or different immune mechanisms are activated in the first versus the second challenge or which mechanisms of the first challenge activate the second challenge remains untested. The references are shown in the supplementary material of our reference 5.

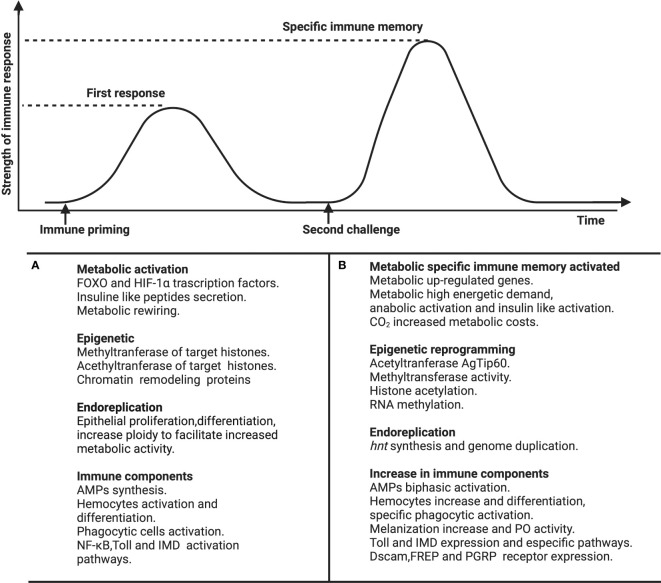

## Introduction

Parasites, virus and pathogens (here referred parasites as the evolutionary strategy) significantly reduce the host fitness. Parasites use hosts as a resource for reproduction, while hosts either eliminate their parasites or undergo infection in one or more occasions throughout their life (1). On the one hand, parasites possess virulence factors that enable them to evade, diminish, or eliminate the host’s immune response, thereby facilitating the establishment of infection (1). On the other hand, the hosts kill or tolerate their parasites through diverse and specialized immune mechanisms to achieve an optimal immune response (1, 2).

A paradigm shift in immunity lies in the invertebrate’s ability to develop specific immune memory (3). The invertebrates immune memory has also been referred to as innate immune memory, alternative immune memory, or immune priming (3–5). This phenomenon is so named to distinguish it from vertebrate adaptive memory, although both can specifically protect the hosts against reinfection in terms of immune response, parasite elimination, and improved survival (4). However, the mechanisms underlying immune memory including storage, maintenance, efectors, recall and the role of factors such as epigenetic modifications in reprogramming remain elusive (5). Additionally, understanding its potential interaction with other physiological pathways such as metabolism is important to understand deeply the immune memory.

## Immunometabolism

**Figure 1:**
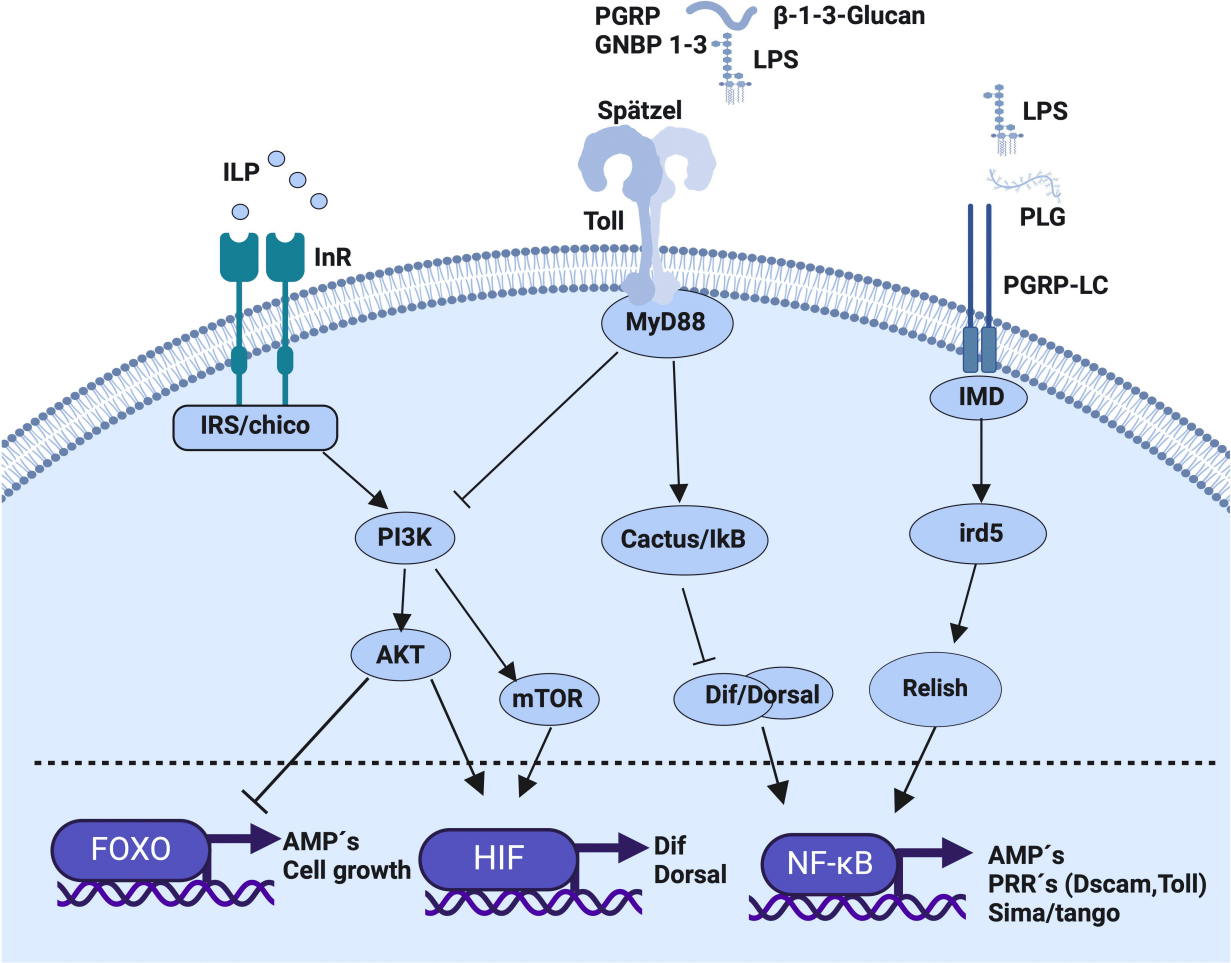
lmmunometabolic relationship established within an immunocompetent invertebrate cell in response to the recognition of metabolic and immune stimuli. Initially, the recognition of insulin-like peptides (ILPs) by the insulin receptor (InR, Chico) activates the insulin signaling pathway (ISP). This activation triggers the PI3K signaling pathway (Phosphoinositide 3-kinase), which, in the case of mTOR activation (mammalian target of rapamycin), culminates in the activation of HIF1α/β (Hypoxia-Inducible Factor 1α/β) for the synthesis of the Dif dimer (Dorsal related immunity factor) and Dorsal (a member of the Rel transcription factor family) to form NF-κB involved in antimicrobial peptide (AMP) synthesis. In the case of AKT pathway activation (Serine-threonine kinase), which inhibits the activity of the FOXO transcription factor (forkhead box; O class transcription factor family), cellular growth, proliferation, differentiation, cellular longevity, and AMP synthesis are inhibited. Subsequently, following Toll receptor activation by the binding of the endogenous cytokine ligand Spätzle (an extracellular ligand of the Toll receptor) recognizing pathogen-associated molecular patterns (PAMPs) from Gram-negative bacteria (LPS-GNBP 1-3; β-1,3-glucan) and Gram-positive bacteria (Lipopolysaccharides bound to PGRP), the MyD88 adapter (myeloid differentiation factor 88) is activated, inducing the degradation of Inhibitor of κB (Cactus in Drosophila), favoring the translocation of the NF-κB nuclear factor and the synthesis of pattern recognition receptors (PRRs) and anti microbial peptide (AMP) genes, while also inhibiting PI3K activity. Finally, the activation of the immunocompetent cell through the Imd pathway (immune deficiency pathway), which activates Ird5 (IkB kinase homologue) and Relish (an NF-κB transcription factor, a key regulator of the Imd pathway) upon binding to PAMPs, LPS, and peptidoglycan through the PGRP-LC receptor (peptidoglycan recognition protein), culminates in NF-κB activation and the synthesis of target genes.

Immunometabolism denotes the intricate interaction between the immune system and the energy acquisition and utilization (6). Some key facets of immunometabolism include: 1) the energy utilization by the immune response; 2) the availability of metabolic energy and 3) the allocation of energy between immunocompetent cells or tissues as well as the trade-off between immune response and life-history traits such as reproduction (4, 6). This allocation is influenced by environmental factors, for example temperature, development, reproduction and maintenance of cellular functions (2, 6–8), and all are tied to immune memory (4, 7–9). In invertebrates, the energy demand during infection is influenced by the parasite (10). Specific metabolic signals are essential for activating and regulating the immune response (**Figure 1**) (6, 11). For example, the insulin pathway is activated in response to infection (12), and metabolic plasticity arises in hemocytes during their differentiation (13). Thus, insulin emerges as a critical molecule in this bidirectional interaction (14). In invertebrates, insulin-like peptides (ILPs) and its receptor (InR) are implicated in metabolism and immunity (**Figure 1**) (15). Activation of InR leads to the phosphorylation of the Akt kinase and the subsequent inactivation of transcription factors such as the subfamily of forkhead box-containing proteins (FOXO) (13) and the TOR (the target of rapamycin) pathway, which represents a nutrient-sensitive signaling cascade crucial for cellular metabolism (**Figure 1**) (16). The InR activation stimulates glucose uptake, lipogenesis, the size and number of mitochondria, and cellular division and differentiation (17, 18). In *Drosophila melanogaster*, the insulin-like peptide and infection are connected: A mutation of the chico gene (insulin receptor homolog), which regulates the signaling pathway dependent on the presence of ILP, increases the survival of flies infected with the Gram-negative *Entomobacter faecalis* and with the Gram-positive *Staphylococcus aureus* (17). This conections are bi-directional in insects because the constitutive activation of the Toll pathway inhibits Akt and triacylglyceride utilization (6). Additionally, the Pathogen-Associated Molecular Patterns (PAMPs), and ILPs, drive the expression of genes involved in the immune response (antimicrobial peptides, cell growth, Dif, Dorsal and pathogen recognition receptors), and factors such as FOXO, NF-κB, and hypoxia-inducible transcription factor (HIF) have a bidirectional communication (**Figure 1**). For example, the transcription factor FOXO downmodulates the cell number and tissue growth in insects by promoting anabolic metabolism (19) and regarding NF-κB, the modulation of the expression of the transcription factors Dorsal, Dif and Relish in *Drosophila* lead to interference between metabolic and immune response pathways. Thus, infection with *E. coli* in *D. melanogaster* that lacks Ird5 (the homologous of IKK, that activates the Relish protein by phosphorilation), produces a decrease in the synthesis of drosomycin and a reduction in the expression of HIF-1α and HIF-1β, genes homologs called sima and tango, respectively (20, 21). Similar alterations occur in mutant flies lacking the sima gene, leading to altered NF-κB function (20). Therefore, oxygen concentrations, energy availability, ILPs, and infection may be interconnected (19).

## The invertebrate’s immune memory

Since the discovery of immune memory in invertebrates, its efficacy has been scrutinized in different host species (4, 5). The effector immune mechanisms (e.g. antimicrobial peptides, pro-oxidants, phagocytosis, or lytic activity) underlying this immune memory show plasticity that may be contingent upon host condition, development, sex, age and/or populations (5). In addition, an important characteristic of immune memory in invertebrates is its ability to be transmitted to subsequent generations, termed transgenerational immune memory (22, 23). Here we are focused only on the specific immune memory within generations. In this topic, two molecular mechanisms are correlated with the invertebrates immune memory: The endocycle and epigenetics and metabolism to a much lesser extent (**Graphical Abstract**).

Hemocytes and epithelial cells are suggested to play a crucial role in establishing immune memory in invertebrates and are closely associated with the endocycle (24, 25). In immune response, the endocycle is triggered in hemocytes after energetic demands, such as viral infections (26, 27). In *An. albimanus* primed with *P. berghei*, a significant upregulation of genes involved in cell cycle elements such as cyclins A, B, E, and factors activating the endoreplication pathway like Notch and Hnt was observed upon subsequent challenge (27, 28) and in the same model system, *An. albimanus* against *P. berghei*, the immune memory group showed more cellular activity of the endocycle than the control group (24, 27). Hence the endocycle seems to be implicated in the invertebrates immune memory.

Epigenetic modifications are crucial in regulating gene expression, thereby influencing host-pathogen interactions. Recent studies have revealed an interaction between immune response and epigenetic modifications, including histone modifications (acetylation, methylation, phosphorylation, and ubiquitination), and chromatin remodeling or structure (29, 30). Few examples have been published regarding specific immune memory and epigenetics whithin generations. DNA methylation is implicated in different biological processes in insects, including host-pathogen interactions (31, 32). In adults and larvae of *T. molitor* infected with *Micrococcus lysodeikticus* or *M. anisopliae*, respectivelly, a differential methylation on RNA during immune memory was identified and no evidence of methylation on DNA was supported (33). Another posttranslational modification of histones is acetylationcatalyzed by histone acetyltransferases (HATs) and histone deacetylases (HDACs) (34). HATs open chromatin structure, promoting access to DNA and gene expression, while the condensed chromatin generated by HDACs leads to gene silencing. This dynamic activity of histone acetylation/deacetylation influences chromatin structure, reflecting the opposing activities of HATs/HDACs. For instance, in *An. gambiae* mosquitoes infected with *P. berghei*, the enzyme histone acetyltransferase AgTip60 was identified as essential in synthesizing hemocyte differentiation factor (HDF), affecting the increase in oenocyte number and maintaining a broad, systemic, and long-lasting state of enhanced immune surveillance in primed mosquitoes (35). During specific immune memory in invertebrates, there seems to be a tight interconnection between the endocycle and epigenetic mechanisms with immune memory. Still, metabolism also appears critical in this interaction (**Graphical Abstract**).

## Immunometabolism in specific immune memory

Certain molecules have been pinpointed in invertebrates, such as the mTOR signaling pathway, which regulates the activation of HIF-1α/β, a conserved mechanism in vertebrates responsible for controlling glucose metabolism (36, 37). Immune stimulation with β-glucans induces alterations in glucose metabolism, including the transition from oxidative phosphorylation to aerobic glycolysis, heightened glutamine metabolism, cholesterol synthesis, and the upregulation of long non-coding RNAs (lncRNAs), mirroring observations in vertebrates (8). Epigenetic modifications, facilitated through posttranslational regulation of enzymes, play a pivotal role in metabolic activation (36). Specific immune memory can prompt the reconfiguration of the immunometabolic network through differential expression of molecules in insect hemocytes. Furthermore, mitochondria are pivotal, housing enzymes responsible for adding or removing epigenetic marks on DNA or histones using metabolites as substrates or co-factors (38). This renders the epigenome susceptible to metabolic changes, such as histone acetylation facilitated by acetyl-CoA (38, 39), which can modulate the activity of histone acetyltransferases (HATs), thus regulating gene expression (40, 41). Another critical metabolic process involves the conversion of pyruvate to mitochondrial oxaloacetate via activation of glutaminolysis, and subsequently to α-ketoglutarate (α-KG) (42), which may be associated with specific immune memory.

Research in *Drosophila melanogaster* has uncovered upregulation of Toll, PGRP, and PRRs during the activation of specific immune memory (43). Concerning the humoral response, some but not all AMPs measured displayed biphasic kinetics in *An. albimanus* against *P. bergei* (24). Finally, activation of immune memory in *Biomphalaria glabrata* triggers a shift from cellular to humoral effector immune responses, accompanied by differential expression of various gene families, including PRRs such as FREP, macrophage lectins, and C-type lectins (44). However, studies like these have not integrated the perspective of immunometabolism, which could elucidate, for instance, whether a shift in immune response is less energetically demanding than a biphasic response. Recent studies have demonstrated that specific immune memory activates the metabolic system (45), and some have explored the components of immunometabolism in invertebrates, comparing the initial immune challenge with the subsequent one (**Graphical Abstract**). Nevertheless, the intricacies of the interaction of metabolism during specific immune memory processes, encompassing recognition, storage, and retrieval, remain incompletely understood (5). While a biphasic response is predicted in immune memory, research in this domain remains limited and biphasic kinetics are not consistently observed (5, 44, 46). Studies following the first challenge have revealed an increase in insulin-like peptides and metabolic rewiring, while after the second challenge, up-regulation of metabolic genes, heightened energetic demand, insulin-like peptide activation, and increased CO_2_ production have been observed (**Graphical Abstract**). How these mechanisms elucidate the utilization of energetic fuel during specific memory and how they explain the evolutionary costs of immune memory remain to be investigated. Additionally, there is plasticity in the kinetics of the immune response from the first to the second challenge, partially explained by the host’s interaction with its environment (5). For instance, temperature, sex, and infections impact metabolism (7, 47, 48), yet there is currently no information on how these variables affect immunometabolism during specific immune memory or how metabolism constrain immune memory based on the organism’s environment.

## The costs of immune memory

The immune response is costly, and hence, the specific immune memory should also be costly (49). This energetic cost can be quantified by assessing changes in the basal metabolic rate (BMR), which denotes the energy expended by an individual at rest, reflected in the amount of CO_2_ exhaled upon activation (50–52). The quantity of CO_2_ emitted could provide insights into the potential costs associated with specific immune memory (7, 52). Notably, parasitism induces a heightened energy demand in the host, increasing BMR (52). In both invertebrates and vertebrates, immune system activation escalates total metabolic rate (53), as energy reserves are mobilized to support immune responses (54, 55) and homeostatic recovery processes (56). Integrating studies on specific immune memory by considering immune and metabolic responses in invertebrates becomes imperative. This holistic approach has shed light on the evolutionary costs associated with specific immune memory in invertebrates and adaptive memory in vertebrates (4).

The activation of specific memory in hosts demands energy, leading to the use of glycogen, lipids, and proteins (57). The magnitude of energy demands dictates the costs associated with specific immune memory activation, evolutionary costs, and their correlation with the biphasic and sustained immune response (4, 5, 7): the higher the energy demand, the greater the cost. Metabolomic analysis will provide information about how the metabolism works to pay the immune memory costs, from the first to the second or subsequent challenges. Future studies could explore whether homologous or heterologous challenges with parasites or pathogens differentially induce evolutionary costs. If immune memory is costly, higher metabolic costs would be predicted in homologous challenges such as dual challenge with a pathogen A than in heterologous challenges conducted against pathogen A after inducing the immune response with pathogen B.

## Future directions to study immunometabolism in invertebrate specific immune memory

One of the most critical aspects that remain understood is the role of epigenetic modifications, such as histone 3, in elucidating the immunometabolism of hemocytes, as well as the epigenetic regulation of metabolic pathways such as the transcription factors HIF-1 and FOXO, and their relevance in the immunometabolic connection of specific immune memory. In invertebrates, posttranslational epigenetic modifications may reconfigure immune and metabolic signaling pathways, the resulting epigenetic landscapes may favor specific responses during immune memory. Additionally, exploring immunometabolism, epigenetics, and the endocycle during intergenerational immune memory could provide insights into its effect on inheritance and its implications for host-parasite dynamics.

We emphazise the need for studying the connections between immune activation, energy expenditure, and metabolic pathways. This could involve examining changes in nutrient utilization, energy allocation, and metabolic rates during homologous *versus* heterologous immune challenges. Of particular interest is the study of how metabolism influences the establishment, storage, and expression of specific immune memory in invertebrates by manipulating metabolic pathways through dietary manipulations or genetic modifications and then, analyzing the resulting impact on immune memory formation and maintenance. Moreover, exploring how metabolic cues influence epigenetic-involved changes in immune gene regulation and immune memory formation may be interesting. This could involve studying changes in DNA methylation, histone modifications, and non-coding RNA expression patterns in response to metabolic perturbations. Exploring the fitness consequences arising from metabolic strategies within the framework of immune defense and pathogen exposure represents a promising avenue of research from an evolutionary standpoint. This research should encompass potential sources of variation that might elucidate the plasticity of specific immune memory, including intrinsic attributes such as developmental stage and sex, as well as extrinsic factors like environmental conditions (temperature) and resource availability. Understanding how variation in metabolism influence the fitness outcomes associated with mechanisms may provide valuable information about the adaptive significance of specific immune memory. Applying a comparative approach to assess evolutionary patterns across diverse invertebrate taxa will provide insights into the macroevolution of the relationship between metabolism and immune memory. By bridging the gap between immunology and metabolism, research has the potential to uncover fundamental principles governing host-pathogen interactions and shape our understanding of evolutionary trade-offs in immune defense.

## Author contributions

TM: Conceptualization, Writing – original draft, Writing – review & editing. JC: Conceptualization, Writing – review & editing. HL: Conceptualization, Writing – review & editing. AO: Conceptualization, Writing – review & editing. KM: Conceptualization, Writing – review & editing. JC-G: Conceptualization, Funding acquisition, Supervision, Writing – original draft, Writing – review & editing.
